# Two identified subsets of CD8 T cells in obstructed kidneys play different roles in inflammation and fibrosis

**DOI:** 10.18632/aging.103764

**Published:** 2020-09-13

**Authors:** Juan Wang, Jijing Tian, Jian Sun, Min Gao, Yanjun Dong

**Affiliations:** 1 College of Veterinary Medicine, China Agricultural University, Beijing 100193, China; 2 Department of Nephrology, Shanghai General Hospital, Shanghai Jiaotong University, Shanghai 200080, China; 3 Animal Husbandry and Veterinary Department, Beijing Vocational College of Agriculture, Beijing 102442, China

**Keywords:** inflammation, fibrosis, CD8 T cells, macrophages, kidney

## Abstract

Inflammation plays a crucial role in initiating renal fibrosis after injury. The infiltration of inflammatory cells, such as CD4^+^ T cells and macrophages, contributes to renal fibrosis following ureteric obstruction. However, the function of CD8^+^ T cells in obstructed kidneys remains unclear. Although CD8^+^ T cell depletion intensifies renal fibrosis by decreasing IFN-γ and increasing IL-4 in the kidneys, the change and role of CD8 T cell populations following environmental changes during renal fibrosis are largely unknown. Here, we identified two CD8 T cell subsets in mouse obstructed kidneys with unilateral ureteric obstruction and revealed their different functions in building an inflammatory or profibrotic environment. Following renal fibrosis, the phenotypes of infiltrated CD8 T cells were mainly Tc1 (CD44^+^CD25^−^CD62L^−^) at the early inflammation stage and then changed to Tc2 (CD44^+^CD25^high^CD62L^low^). Tc1 and Tc2 secreted IFN-γ, contributing to the decrease in the Th2-induced over-polarization of M2 macrophages and fibrosis. Moreover, Tc2 secreted pro- and anti-inflammation factors and decreased the inflammatory responses of other cells to control inflammation and fibrosis. This work and our previous study showed that CD8 T cells could balance out inflammation by controlling its level in renal fibrosis.

## INTRODUCTION

Inflammation plays a crucial role in initiating renal fibrosis after injury [1]. The peritubular infiltration of inflammatory cells determines the extent and duration of renal interstitial fibrosis following obstructive injury [2]. T lymphocytes are predominant immune cells that infiltrate kidneys after obstructive injury [3]. The recruitment and activation of T lymphocytes typically precede the influx of macrophages into the injured kidneys [4]. T cells have been detected in the kidneys of patients with chronic kidney disease [5] and in the models of renal fibrosis, such as unilateral ureteric obstruction (UUO) [6–10]. However, the role of specific T cell populations in tubulointerstitial fibrosis and the mechanistic details remain unknown.

CD8^+^ T cells are usually cytotoxic T lymphocytes that respond to antigenic challenge through the lysis of their target cells, and CD4^+^ T cells are helper cells that produce lymphokines and play a role in the activation and phenotypic switch of macrophages [11]. CD8^+^ T cells are cytotoxic and can secrete inflammatory factors to regulate other inflammatory cells [12]. The depletion of CD8^+^ T cells increases renal fibrosis following ureteric obstruction, and IFN-γ expresses CD8^+^ T cells [13] and CD11c^+^CD8^+^ T cells [14] and contribute to this process through different mechanisms. Similar to CD4^+^ T cells that switch subsets from Th1 to Th2 in the late inflammation stage [15], CD8^+^ T cells may switch the subsets following the exacerbation of inflammation to play different roles in the profibrotic microenvironment. The expression of several important surface adhesion molecules mediating extravasation and subsequent lymphocyte trafficking to sites of inflammation can be used to identify the phenotypes of resting and activated T cells in infectious diseases; CD44 and CD25 are activated T cell markers, and CD62L is a resting T cell marker [16]. CD62L downregulation promotes the extravasation of lymphocytes to inflammation sites, CD44 expression in activated lymphocytes promotes their movement through the extracellular matrix via interactions with hyaluronic acid and fibronectin, and highly activated T cell populations express high CD25 levels [17]. The existence and function of CD8 T cell subsets identified by these key markers exist following renal inflammatory development in renal fibrosis remain unknown.

Renal inflammation is promoted by various inflammatory cells, but its progression to fibrosis depends on macrophages transition from a pro-inflammatory M1 phenotype to a profibrotic M2 phenotype; these macrophages and other cells then build a profibrotic microenvironment to promote fibroblast differentiation into collagen-producing myofibroblasts [18]. In this study, different CD8 T cell subsets in the obstructed kidneys of UUO mice were identified by using CD44, CD25, and CD62L at days 0, 3, 5 and 7 post-UUO and then isolated to examine their secretion capability for inflammatory factors by applying a test kit for 23 inflammatory factors. CD8 T cell subsets were co-cultured with macrophages to test the M2 maker (Arg-1 and CD206) in cells and with inflammatory factors in a medium to examine their effects on macrophage transition. The CD8 T cell subsets were also co-cultured with macrophages and fibroblasts in a medium to compare their differences in myofibroblast marker (a-SMA, collagen, and fibronectin) expression levels, fibroblast contractibility, and inflammatory factors and to examine fibroblast differentiation in the microenvironments built by CD8 T cell subsets and macrophages.

## RESULTS

### Renal fibrosis in UUO mice was accompanied by the infiltration of two different CD8 T cell subsets

**Figure 1:**
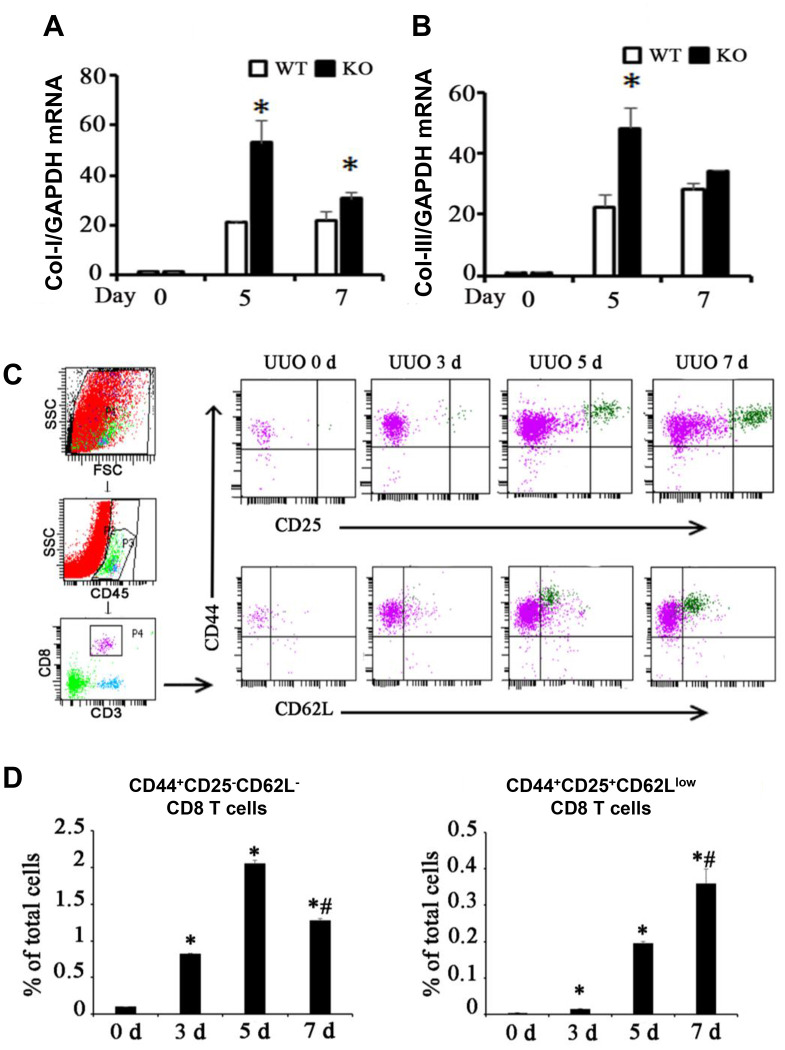
**Two different subsets of infiltrating CD8 T cells were related to renal fibrosis in UUO model mice.** (**A**, **B**) Total mRNA was obtained from the UUO kidneys of WT mice, CD8 KO mice, or CD8 KO mice transplanted with CD8 T cells at days 0, 5, and 7. The results showed the expression levels of Col-I and Col-III in CD8 KO mice compared with those in WT mice. (*p < 0.05 vs. WT UUO). (**C**) Representative examples of FACS analysis at each point. CD8^+^ T cells in obstructed kidneys were identified on the basis of CD45, CD3, CD44, CD25, and CD62L expression by using flow cytometry. (**D**) CD44^+^CD25^−^CD62L^−^ and CD44^+^CD25^+^CD62L^low^ CD8^+^ T cells were counted and analyzed at days 0, 3, 5, and 7 after UUO (*p < 0.05 vs. 0d, #p < 0.05 vs. 5d).

CD8^+^ depletion intensified renal fibrosis [13, 14] as shown in Figure 1A, 1B. CD44 and CD25 are markers of activated T cells, andCD62L is a marker of resting T cells. Two different CD8^+^ T cell subsets were found in the kidneys following UUO (Figure 1C): one is the major population marked by CD44^+^CD25^−^CD62L^−^ (Subset 1, Tc1), and the other is a minor population marked by CD44^+^CD25^high^CD62L^low^ (Subset 2, Tc2). Tc1 appeared earlier and more abundantly than Tc2 (Figure 1D). These results showed that following renal fibrosis, the infiltrated CD8 T cells changed their subset from Tc1 (CD44^+^CD25^−^CD62L^−^) to Tc2 (CD44^+^CD25^high^CD62L^low^).

### Tc2 exhibited higher secretory capability than Tc1 especially for anti-inflammatory factors

**Figure 2:**
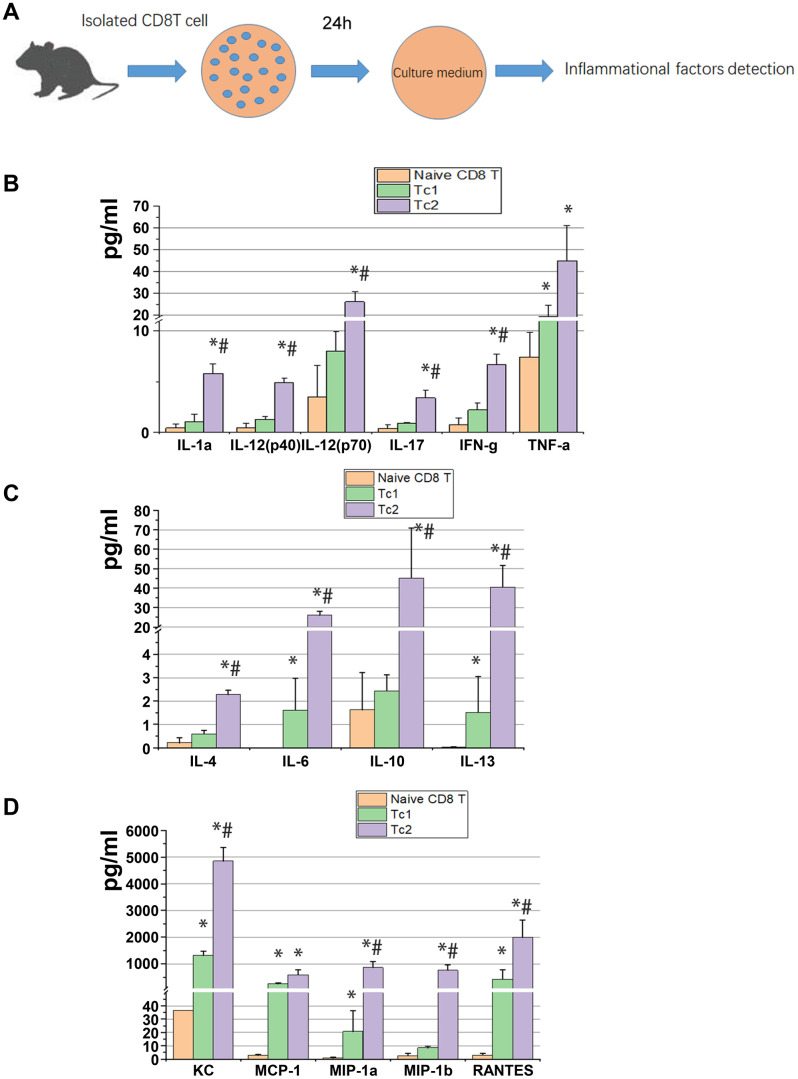
**Tc2 facilitated the secretion of cytokines, especially anti-inflammatory factors, compared with Tc1.** (**A**) Naïve CD8^+^ T cells (CD44^−^CD25^−^CD62L^high^) from the spleens of WT mice and Tc1 and Tc2 from 7-day UUO kidneys were isolated and cultured for 24 h (2 × 10^5^ cells per well). The culture medium was collected for the detection of inflammatory factors by using a Luminex multiplex murine cytokine assay. (**B**–**D**) Proinflammatory cytokines, anti-inflammatory cytokines, and chemokines in the cell culture medium that were significantly changed are shown (*p < 0.05 vs. naïve CD8^+^ T cells, #p < 0.05 vs. Tc1).

At day 7 after UUO were cultured *in vitro* for 24 h, 2 × 10^5^ cells/well of Tc1 and Tc2 were isolated from the obstructed kidneys, and non-activated CD8 T cells (CD44^−^CD25^−^CD62L^high^) were isolated from spleens as the control (Figure 2A). The 23 factors related to inflammation were tested, and the results showed that 15 factors changed among the three subsets. These 15 factors were more elevated in Tc1 and Tc2 in medium than in non-activated CD8 T cells, and the secretory capability of Tc2 was stronger than that of Tc1 (Figure 2B–2D). Tc2 secreted pro-inflammatory factors (IL-1a, IL-2, IL-17, INF-γ, and TNF-a) and chemokines (KC, MCP-1, MIP-1α, MIP-1β, and RANTES) by several folds and anti-inflammatory factors (IL-4, IL-10, and IL-13) and IL-6 by more than 10-fold compared with Tc1. These phenomena occurred after the renal inflammation CD8 T cells developed toward an anti-inflammatory phenotype.

### Tc2 showed stronger capability for inducing macrophage development to M2 than Tc1

**Figure 3:**
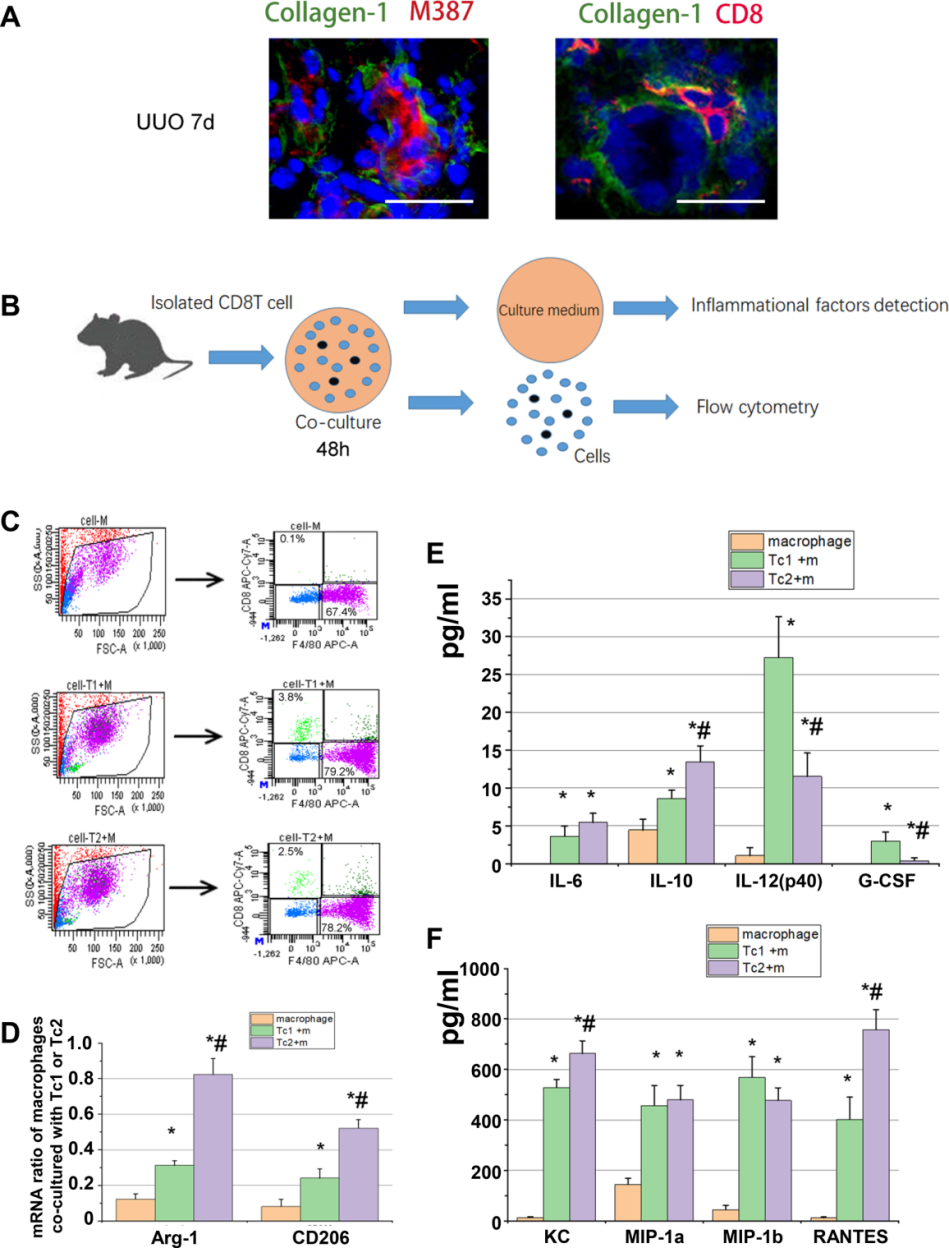
**Tc2 showed stronger capability for inducing macrophage development to M2 than Tc1.** (**A**) Representative photomicrographs showing kidney sections from UUO mice at day 7. The sections were stained with Collagen-1 (green) and M387 or CD8 (red), counterstained with DAPI (blue), and examined through confocal microscopy (scale bars, 20 μm). Positive signals were observed in the renal interstitium. (**B**) CD8^+^ T cells (Tc1 and Tc2) were isolated from the kidneys of UUO mice and cocultured with Raw264.7 cells for 48 h (1 × 10^4^ T cells and 1 × 10^5^ Raw264.7 cells per well). The cell culture medium was collected for inflammatory factor detection, and the cells were collected for flow cytometry. (**C**) Representative examples of the FACS analysis of cocultured cells. The cells were stained with CD8 and F4/80, and Raw264.7 cells were sorted through flow cytometry for mRNA examination. (**D**) mRNA levels of Arg-1 and CD206 in M2 were tested by using qPCR (*p < 0.05 vs. Raw264.7 cells, #p < 0.05 vs. Tc1 + Raw264.7 cells). (**E**, **F**) Inflammatory factors were evaluated by using a Luminex multiplex murine cytokine assay. Cytokines that were significantly elevated are shown (*p < 0.05 vs. Raw264.7 cells, #p < 0.05 vs. Tc1 + Raw264.7 cells).

IL-4, IL-10, IL-13, and IL-6 are key signals for macrophage differentiation to the M2 phenotype. In the obstructed kidneys, CD8 T cells and macrophages (M387, a macrophage marker) were located with collagen-1 in an interstitial region (Figure 3A). CD8 T cells were co-cultured with RAW264.7 cells to test the levels of M2 marker (Arg-1 and CD206) and inflammatory factors in the medium (Figure 3B) and determine whether the different CD8 T cells subsets affect macrophage phenotype and inflammatory factor secretion. After 48 h of culture, the macrophages were separated from each group as shown in Figure 3C, and the relative mRNA expression of M2 was measured. The results showed that the Tc2-treated macrophages elevated Arg-1 and CD206 compared with the Tc1-treated macrophages (Figure 3D). Chemokine secretion (KC, MIP-1a, MIP-2b, and RANTES), inflammatory factor levels (IL-6, IL-10, and IL12), and G-CSF were elevated in the Tc1- and Tc2-treated macrophages. The Tc2-treated macrophages showed higher IL10 and lower IL-12 and G-CSF levels than the Tc1-treated macrophages (Figure 3E, 3F). These results indicated that CD8 T cells activated macrophage development and promoted inflammatory cell recruitment through the action of chemokines. Moreover, Tc2 exhibited stronger inducing capability for macrophage development toward M2 than Tc1.

### Tc1-treated macrophages activated fibroblasts to secrete inflammatory factors, and Tc2-treated macrophages promoted fibroblast differentiation into myofibroblasts

**Figure 4:**
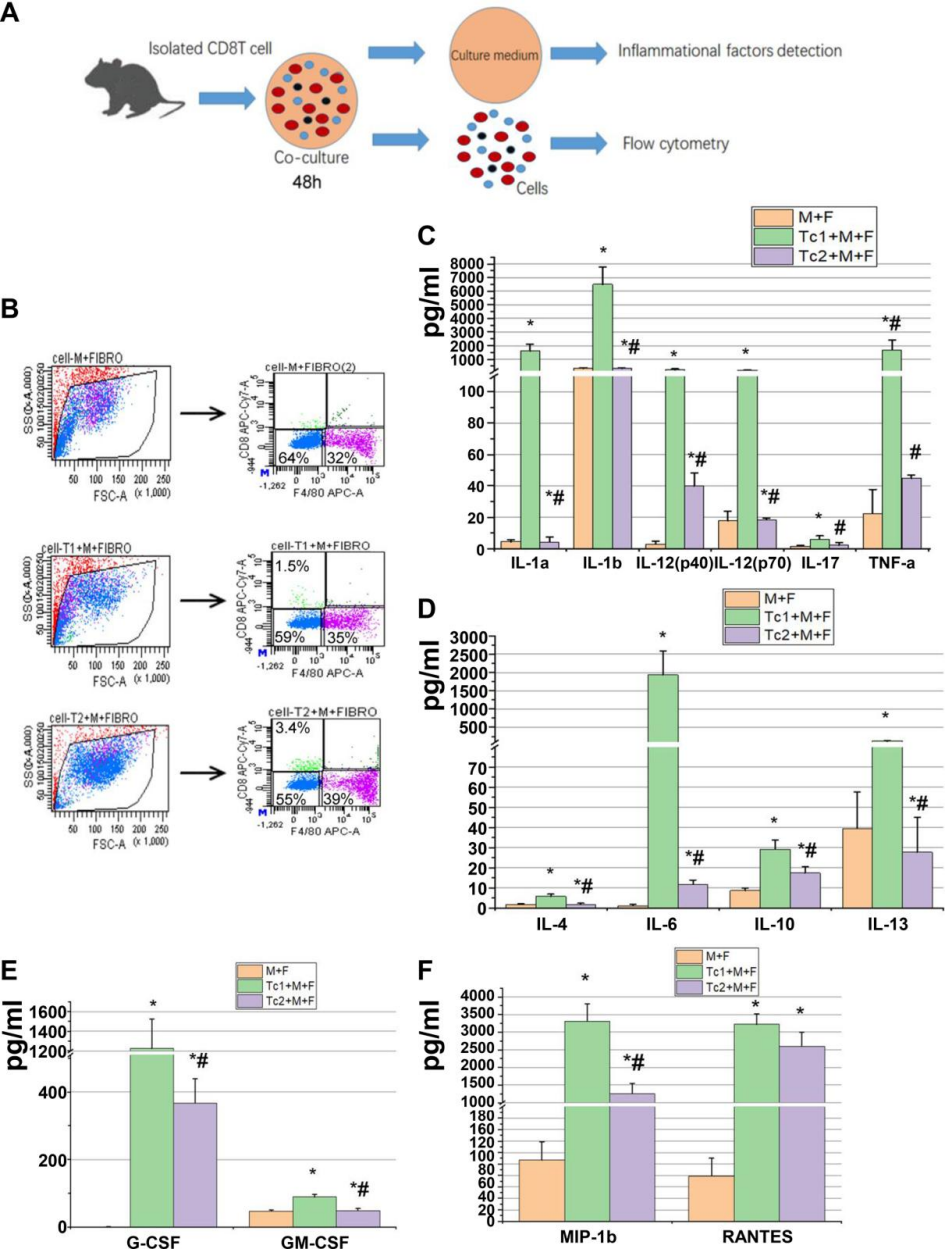
**Tc1-treated macrophages promoted the activation of fibroblast to secrete inflammatory factors.** (**A**) CD8^+^ T cells (Tc1 and Tc2) were isolated from UUO kidneys and cocultured with Raw264.7 cells plus NIH3T3 cells for 48 h (1 × 10^4^ T cells, 1 × 10^5^ Raw264.7 cells, and 2 × 10^5^ NIH3T3 cells per well). Cell culture medium was collected for inflammatory factor detection, and cells were collected for flow cytometry. (**B**) Representative examples of the FACS analysis of cocultured cells. Cells were stained with CD8 and F4/80 and sorted through flow cytometry; then, NIH3T3 cells (CD8^−^F4/80^−^ cells) were collected for the examination of α-SMA, Col-1, and fibronectin mRNA (data shown in Figure 5). (**C**–**F**) Inflammatory factors were evaluated by using a Luminex multiplex murine cytokine assay, and those that were significantly elevated are shown (*p < 0.05 vs. Raw264.7 cells + NIH3T3 cells, #p < 0.05 vs. Tc1 + Raw264.7 cells+ NIH3T3 cells).

**Figure 5:**
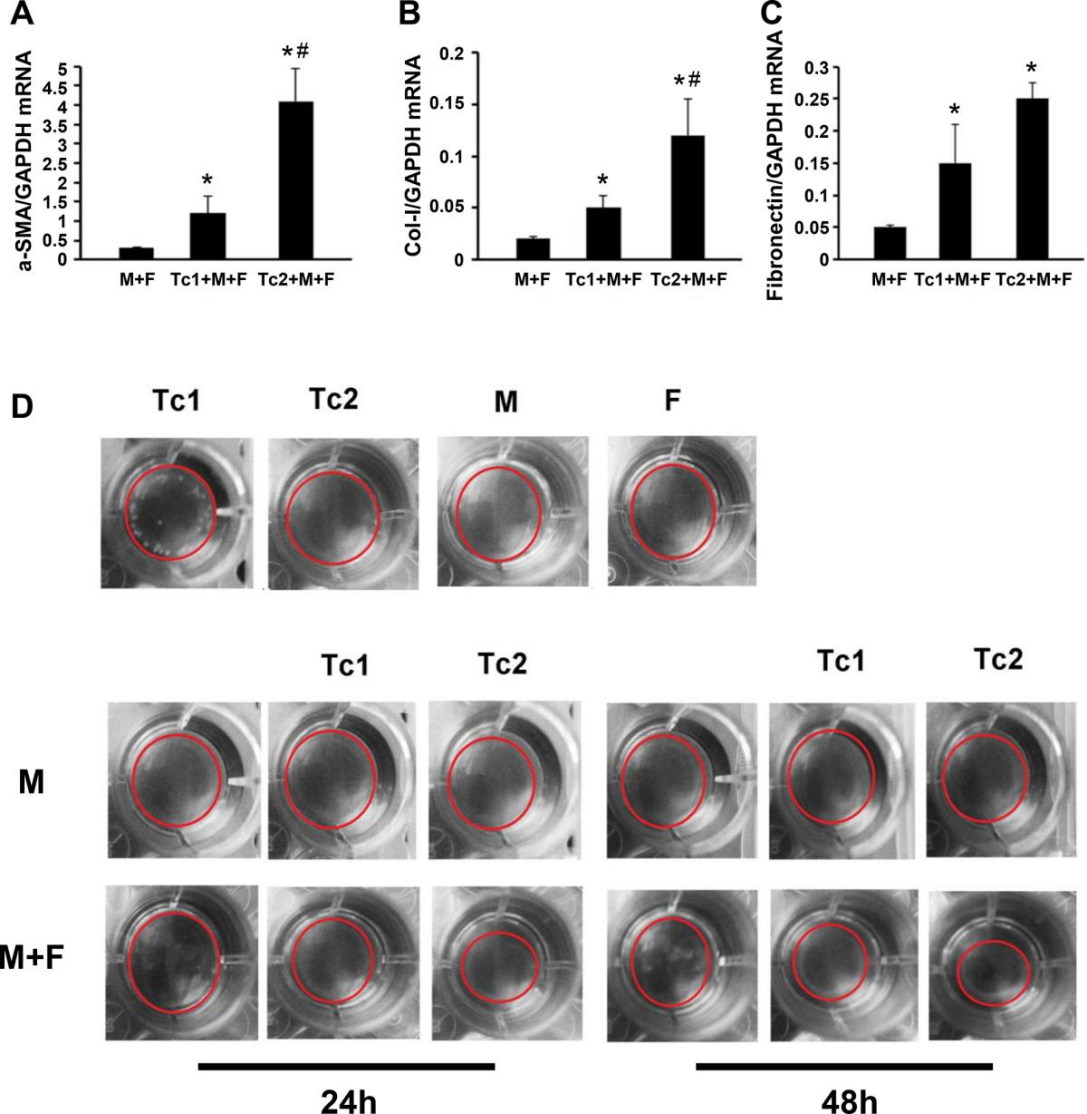
**Tc2-treated macrophages promoted the differentiation of fibroblasts to myofibroblasts more than Tc1.** (**A**–**C**) NIH3T3 cells cocultured with Raw264.7 cells, Tc1 plus Raw264.7 cells, or Tc2 plus Raw264.7 cells were collected through cell sorting for mRNA examination (*p < 0.05 vs. Raw264.7 cells + NIH3T3 cells, #p < 0.05 vs. Tc1 + Raw264.7 cells + NIH3T3 cells). (**D**) NIH3T3 cells were cocultured for 24 and 48 h with Raw264.7 cells plus Tc1 or Tc2 to assay cell contraction assay by using a cell contraction assay kit. Changes in gel size were indicated by red circles.

M2 macrophage-induced fibroblast differentiation to myofibroblasts is a key mechanism of renal disease progressing from a predominantly pro-inflammatory phase to a chronic fibrotic stage. CD8, RAW264.7, and NIH3T3 cells (a fibroblast cell line) were co-cultured for 48 h to test the phenotypes of fibroblasts induced by the macrophages treated with different CD8 T cell subsets. Fibroblasts were separated by FACS to examine the relative mRNA expression levels of myofibroblast markers and test the inflammatory factors in the culture medium (Figure 4A, 4B). The fibroblasts were responsive to Tc1-treated macrophages and secreted higher levels of pro-inflammatory factors compared with macrophage-induced fibroblasts or Tc2-treated macrophages (Figure 4C–4F). The mRNA expression levels of myofibroblast markers in fibroblasts showed that the Tc2-treated macrophages had significantly increased a-SMA and Col-1 mRNA expression levels (Figure 5A–5C). Myofibroblasts exhibited stronger contraction than fibroblasts. Cell contraction was tested through cell contraction assay to confirm whether Tc2-treated macrophages promote fibroblast differentiation to myofibroblasts. After 24 or 48 h of culture, the collagen size of the Tc2-treated macrophages was smaller than that of the other groups (Figure 5D). These results indicated that different fibroblasts phenotypes were induced by Tc1- or Tc2-treated macrophages. The former activated fibroblasts to secrete inflammatory factors, and the latter promoted fibroblast differentiation into myofibroblasts, thereby decreasing inflammatory response and secreting additional ECM.

## DISCUSSION

Tubulointerstitial fibrosis degree predicts the long-term outcome of renal function [19, 20]. Inflammatory cells, such as T cells [10], macrophages [21], and neutrophils [22], are involved in tissue fibrosis. Although widely studied as cytotoxic cells in immunology, the function of CD8^+^ T cells in non-immune inflammatory diseases remains unclear. CD8 T cell depletion intensifies renal fibrosis following ureteric obstruction [13, 14]. Here, IFN-γ-expressing CD8 T cells were found to impair CD4 Th2 cell accumulation to contribute to this process. If IFN-γ (a pro-inflammatory factor) secretion is the only function of CD8 T cells in UUO-induced renal fibrosis, then the RAG^−/−^ mice (neither CD4 T cells nor CD8 T cells) reconstituted with CD8^+^ T cells would experience more mild inflammation and fibrosis compared with RAG^−/−^ mice. However, none of the fibrosis markers showed substantial differences between RAG^−/−^ mice (neither CD4 T cells nor CD8 T cells) reconstituted with or without CD8^+^ T cells [10]. This conclusion was previously confirmed by depleting CD4^+^ T cells in CD8 KO mice and CD8^+^ T cell-reconstituted CD8 KO mice. Following the progress of inflammation, any alteration in the microenvironment promotes the phenotype change of CD8 T cells closer to those of CD4 T cells in obstructed kidneys. The different phenotypes of CD8 T cells contribute to building inflammatory or fibrotic microenvironments. On the basis of this result, the phenotypes of CD8 T cells at different stages of UUO-induced fibrosis were determined, and the differences in inflammatory factor levels and fibrosis promotion between the identified CD8 T cell subsets were examined.

During immune response to a viral infection, primed CD8^+^ T effectors exhibit upregulated CD44, downregulated CD62L, and high CD25 levels, whereas naive CD8^+^ T cells exhibit CD44^low^, CD62L^high^, and CD25^−^. Furthermore, resting memory cells present CD44^high^, CD62L^high^, and CD25^−^ [23]. Two different subsets of CD8^+^ T effectors were found in the obstructed kidney after UUO: the first consists of CD44^+^, CD25^−^, and CD62L^−^ (Subset 1, Tc1) and the other includes CD44^+^, CD25^+^, and CD62L^low^ (Subset 2, Tc2). CD8^+^ T cells are required in increasing the expression of chemokines and cytokines [24] and the infiltration of inflammatory cells [12, 25]. Consistent with the mRNA expression of isolated total CD8^+^ T cells in obstructed kidneys [14], our present results showed that Tc1 and Tc2 secreted chemokines (such as KC, MCP-1, MIP-1α, MIP-1β, and RANTES) as illustrated in Figure 2D. In addition, Tc1 and Tc2 could enhance the secretion of several chemokines by macrophages and fibroblasts (Figure 3F), but a deficiency in CD8 did not affect the expression of MCP-1, MIP-1α, MIP-1β, and RANTES in obstructed kidneys [13]. These results indicated that CD8 T cells contributed to chemokine accumulation in obstructed kidneys, but other secretory cells could compensate for its absence.

In the UUO model, inflammatory cell infiltration after injury induces a pro-inflammatory environment and enhances macrophage recruitment in the kidneys; the macrophages then switch to an anti-inflammatory phenotype [26]. The transition from pro-inflammatory to anti-inflammatory environment is a key event. Inflammation mainly progressed at UUO 0–7 days, during which the infiltrated CD8 T cells changed their subset from Tc1 to Tc2. The peak level of Tc2 was not determined. In early UUO stage, inflammatory cell infiltration progressed, and a high peak occurred from days 3-7, including CD8 T cells. The total number of CD8 T cells was reduced at 7 days after surgery, and the accumulated M2 macrophage-induced myofibroblasts secreting ECM is the main mechanism of renal fibrosis. CD8^+^ T cells mainly contribute to building profibrotic environment during the early UUO stage; hence, experiments were performed at days 0-7 post-UUO. Anti-inflammatory factor (IL-4, IL-10, and IL-13) and IL-6 secretion by Tc2 was 10-fold higher than that by Tc1, although the pro-inflammatory factor secretion by Tc2 increased by several folds. Therefore, as a balancer of inflammation, CD8 T cells controlled the level of inflammation, and Tc1 contributed to pro-inflammation and over-inflammation at the early stage of injury and then activated Tc2 and Th2 (a key CD4 subset for strongly inducing an anti-inflammatory and profibrotic environment). Tc2 secreted anti-inflammatory factors to control inflammation and impaired the overdifferentiation of CD4 T cells to Th2 by increasing IFN-γ release to prevent the rapid construction of the profibrotic environment. A study isolated CD8^+^ T cells from IFN-γ KO mice and used them or WT CD8^+^ T cells to reconstitute CD8 KO mice and reported that IFN-γ-CD8^+^ T cells impair the differentiation of CD4^+^ T to Th2 cells and renal fibrosis. Reconstitution by IFN-γ KO CD8^+^ T cells did not reduce fibrosis or impair CD4^+^ T cell differentiation to a Th2 phenotype. By contrast, reconstitution with WT CD8^+^ T cells reduces fibrosis and impairs CD4^+^ T cell differentiation to a Th2 phenotype. In the present work, Tc1 and Tc2 secreted IFN-γ and possibly inhibited the differentiation of Th2 cells and further prevented the Th2 cell-induced polarization of M2 cells. The enhanced pro- and anti-inflammatory factor release by Tc2 could explain why the inflammation and fibrosis caused by IFN-γ-expressed CD8 T cells in RAG^−/−^ mice reconstituted with CD8^+^ T cells were not more severe than those in RAG^−/−^ mice.

Following the progression of renal inflammation to a profibrotic environment accompanied by increased Tc2 infiltration and decreased Tc1 infiltration, the Tc1-treated macrophages activated the fibroblasts to secrete inflammatory factors, and the Tc2-treated macrophages promoted fibroblast differentiation to myofibroblasts. Macrophage differentiation is regulated by the profibrotic cytokine IL-4 and is inhibited by the antifibrotic cytokine IFN-γ, which is primarily produced by CD4^+^ Th1 cells or CD8^+^ T cells during fibrosis regulation after injury. In accordance with the function of IFN-γ in macrophage differentiation, CD8^+^ cells should keep macrophages in an inflammatory phenotype (M1). In the present work, either Tc1 cells or Tc2 cells promoted M2 differentiation *in vitro*, but Tc2-treated macrophages showed highly expressed M2-related genes and secreted highly anti-inflammatory factors compared with Tc1-treated macrophages because of their ability to secrete Th2 cytokines. Macrophages are mainly activated by IL-4 and IL-13, both Th2 cytokines, to facilitate M2 macrophage differentiation and repair. Activated M2 macrophages directly promote myofibroblast proliferation and survival by producing profibrotic factors. Macrophage-myofibroblast transition (MMT) contributes to renal fibrogenesis through M2 macrophage polarization. Here, Tc2 induced M2 macrophage polarization and thus contributed to MMT. Th2 cells are the main promoter for M2 differentiation in renal fibrosis, and the absence of CD4^+^ T cells CD8^+^ T cells neither elevate M2 macrophages nor increase kidney fibrosis for UUO mice *in vivo*. Although CD8^+^ T cells promoted M2 differentiation *in vitro*, they were not able to change the macrophage phenotype by themselves *in vivo*. M2 macrophages in renal fibrosis were decreased by the INF-γ produced by CD8^+^ T cells via inhibiting the differentiation of CD4^+^ T cells to Th2 cells. Although Tc2 cells could induce M2 macrophages and myofibroblasts *in vitro*, this is not their main function in renal fibrosis. CD8 depletion enhances UUO-induced fibrosis, and the reconstitution of RAG^−/−^ mice with CD8^+^ T cells does not induce any changes in fibrosis.

**Figure 6:**
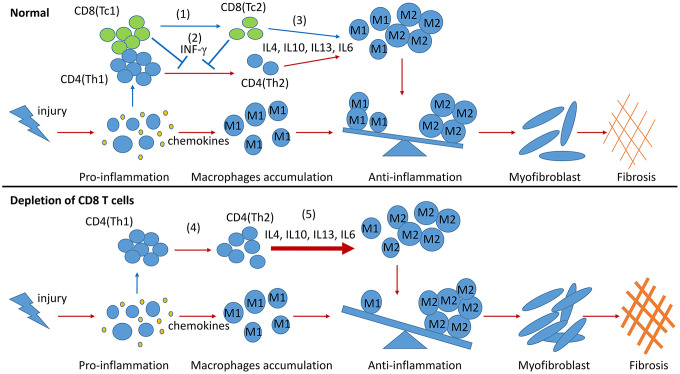
**Model summarizing the role of CD8 T cells in renal inflammation and fibrosis response to UUO.** During the process of renal fibrosis, two subsets of CD8 T cells collaborate with each other to control the progression of inflammation from proinflammation to anti-inflammation and avoid excessive renal fibrosis. The transformation of Tc1 cells to Tc2 cells facilitates the conversion of proinflammation to anti-inflammation after renal injury (1). Tc1 and Tc2 cells secrete INF-γ to inhibit the differentiation of Th2 cells and to prevent the Th2 cell-induced excessive polarization of M2 cells (2). The anti-inflammatory factors secreted by Tc2 cells can also promote the polarization of M2 cells. M2 cells further participate in the construction of a profibrotic environment (3). CD8 T cell depletion results in the accumulation of Th2 cells (4), leading to the excessive polarization of M2 cells, which further exacerbates renal fibrosis (5).

In summary, two CD8 T cell subsets were identified in the obstructed kidneys of UUO mice, and their different functions in building an inflammatory or profibrotic environment were revealed. Following renal fibrosis, the infiltrated CD8 T cells were mainly Tc1 (CD44^+^CD25^−^CD62L^−^) at the early inflammation stage. These cells then changed their phenotypes to Tc2 (CD44^+^CD25^high^CD62L^low^). Tc1 and Tc2 secreted IFN-γ, contributing to the decrease in the Th2-induced over-polarization of M2 macrophages and fibrosis. Moreover, Tc2 secreted pro- and anti-inflammatory factors and decreased the inflammatory response of other cells to control inflammation and fibrosis (Figure 6). This work and our previous study indicated that CD8 T cells could balance out inflammation by controlling its level in renal fibrosis.

## MATERIALS AND METHODS

### Animals and surgery

Male CD8^−/−^ mice and WT littermates with a C57BL/6 background were purchased from Jackson Laboratory (Bar Harbor, ME). All mice used for experiments were 10–12 weeks of age, maintained under specific pathogen-free conditions in the Laboratory of Animal Experiments at Capital Medical University, given a standard diet, and subjected to UUO [19] under ketamine/xylazine anesthesia. A midline incision was made, and the left ureter was exposed and tied off. Sham surgery was performed similarly but without ureter ligation. The mice were sacrificed on postoperative days 0, 1, 3, 5, or 7 through cardiac exsanguination. Kidneys were collected for analyses. All animal care and experimental protocols followed the Animal Management Rule of the Ministry of Health, People’s Republic of China (Documentation no. 55, 2001) and the Guide for the Care and Use of Laboratory Animals published by the United States National Institutes of Health (NIH Publication no. 85-23, revised 1996) and were approved by the Animal Care and Use Committee of China Agricultural University.

### Histology and imaging

After anesthesia was administered, the mice were perfused with PBS through the left ventricle, and the kidneys were removed and processed for cryosectioning or paraffin sectioning. Immunofluorescence was performed using antibodies against CD8 (Becton Dickenson, Franklin Lakes, NJ), collagen-1 (Abcam, Cambridge, MA), and M387 (Abcam, Cambridge, MA). The sections were permeabilized with 0.3% Triton X-100 in PBS, blocked with protein block (DAKO, Glostrup, Denmark) for 1 h at room temperature, and incubated with a primary antibody mixed in antibody dilution (DAKO). The primary antibodies were detected by using second antibodies conjugated to Alexa 568 or 488 (Invitrogen, Eugene, OR). The tissues were visualized with a Nikon 80i microscope, and images were acquired using a DS-cooled camera and NIS-Elements Br 3.0 software (Melville, NY).

### RT-PCR analysis

RNA was extracted through the Trizol reagent method (Invitrogen). Aliquots of 2 μg of total RNA were used for first-strand cDNA synthesis with Moloney murine leukemia virus reverse transcriptase (Promega, Southampton, UK). Aliquots of 2 μL of reaction mixture were amplified with 10 μL of SYBR Green PCR Master Mix (Kangwei, Beijing, China) and 1 μmol/L primers.

Amplification was performed at 95 °C for 5 min, 95 °C for 5 s, and 60 °C for 30 min for each step for 45 cycles. Relative gene expression was calculated from cycle threshold (Ct) values by using GAPDH as an internal control (relative expression = 2^(sample Ct − GAPDH Ct)^). All samples were run in duplicate. The primers and their sequences are listed in Table 1.

**Table 1:** RT-PCR primer sequences.

**mRNA**	**Forward**	**Reverse**
***Col-1***	5’-GCTGGTCTTCCAGGTCCTAAG-3’	5’-CGCCATCTTTGCCAGGAGAA-3’
***Fibronectin***	5’-AGCCCGGATGTCAGAAGCTATAC-3’	5’-AGCGTGTACAGGTGGATCTTG-3’
***Col-III***	5’-CTGTAACATGGAAACTGGGGAAA-3’	5’-CCATAGCTGAACTGAAAACCACC-3’
***a-SMA***	5’-ACTGGGACGACATGGAAAAG-3’	5’-CATCTCCAGAGTCCAGCACA-3’
***Arg1***	5’-AACAC-GGCAGTGGCTTTAAC-3’	5’-GAGGAGAAGGCGTTT-GCTTA-3’
***CD206***	5’-CTCTGTTCAGCTATTGGACGC-3’	5’-CGGAATTTCTGGGATTCAGCTTC-3’
***GAPDH***	5’-TGCCCCCATGTTTGTGATG-3’	5’-TGTGGTCATGAGCCCTTCC-3’

### Tissue preparation, fluorescence-activated cell sorting (FACS), and flow cytometry

Kidneys and spleens were dissected and ground separately. The kidney fragments were digested with 2 mL of collagenase type IA (2.5 U/mL, Sigma Chemical Company, St. Louis, MO) in PBS with 10 mM CaCl_2_ at 37 °C for 30 min. After washing, the kidney and spleen slurries were passed separately through a 40 μm strainer (Becton Dickenson, Franklin Lakes, NJ) and washed with PBS. Cells were collected through centrifugation at 1500 rpm for 5 min, incubated in PBS containing 2 mM EDTA and 2% FBS plus primary antibodies for 30 min at 4 °C, and resuspended at approximately 1 × 10^7^ cells/mL prior to sorting or analysis. The cells were then separated and analyzed by the China Agricultural University Cytometry and Cell Sorting Core Facility by using BD FACSAria II or BD FACSCanto II. The antibodies used were anti-CD45-V500, anti-CD3e-PE-cf594, anti-CD62L-PE, anti-CD8-allophycocyanin-Cy7, anti-F4/80-allophycocyanin, and anti-CD44-FITC. Staining was performed in accordance with the standardized protocol of the BD Company.

### Multiplex measurements

Three subsets of CD8^+^ T cells (non-activated cells originated from the spleen and two subsets of activated cells were obtained from obstructed kidneys 7 days after UUO) collection and cultured in 24-well plates (2 × 10^5^ cells/well) for 24 h or co-cultured with macrophages (1 × 10^4^ T cell cells and 1 × 10^5^ macrophages) for 48 h or with T cells (1 × 10^4^), macrophages (1 × 10^5^), and fibroblasts (2 × 10^5^) in a well for 48 h. Conditional media were analyzed for IL-1, IL-2, IL-3, IL-4, IL-5, IL-6, IL-9, IL-10, IL-12, IL-13, IL-17, Eotaxin, G-CSF, GM-CSF, IFN-γ, KC, MCP-1, MIP-1, and RANTES protein levels by using a Luminex multiplex murine cytokine assay kit (Bio-Rad, Beijing, China) in accordance with the manufacturer’s instructions. Data were analyzed using Bio-Plex Manager software (Bio-Rad^TM^ 200 System).

### Cell contraction assay

A cell contraction assay kit (Cell Biolabs, Beijing, China) was used to test fibroblast contraction in accordance with the manufacturer’s instructions. The cells were harvested and resuspended in the desired medium at 3 × 10^6^ cells/mL. A collagen lattice was then prepared by mixing two parts of the cell suspension and eight parts of cold collagen gel solution. In brief, 0.5 mL of the cell–collagen mixture was added to each well of a 24-well plate, incubated for 1 h at 37 °C, and added with 1 mL of culture medium. After 24 or 48 h, the cells were treated with 1 mM BDM for 30 min. Changes in gel size were compared 1 h after the stressed matrixes were released.

### Statistical analysis

Data were expressed as mean ± standard error of the mean and compared across different mouse strains and time points by using two-way ANOVA. Significance testing was performed using one-way ANOVA, followed by pair-wise comparisons using the Student–Newman–Keuls test. Statistical significance was set at p < 0.05. A minimum of five replicates were performed for each experimental condition.
